# Validation of a one year fracture prediction tool for absolute hip fracture risk in long term care residents

**DOI:** 10.1186/s12877-018-1010-1

**Published:** 2018-12-27

**Authors:** Ahmed M. Negm, George Ioannidis, Micaela Jantzi, Jenn Bucek, Lora Giangregorio, Laura Pickard, John P. Hirdes, Jonathan D. Adachi, Julie Richardson, Lehana Thabane, Alexandra Papaioannou

**Affiliations:** 1 Geriatric Education and Research in Aging Sciences (GERAS), St Peter’s Hospital, 88 Maplewood Ave, Hamilton, ON Canada; 2 School of Rehabilitation Sciences, IAHS 403, McMaster University, 1400 Main St. W., Hamilton, Ontario L8S 1C7 Canada; 3 Department of Medicine, McMaster University, Hamilton, Ontario Canada; 4 School of Public Health and Health Systems, University of Waterloo, Waterloo, Ontario Canada; 5 Department of Kinesiology and Schlegel-UW Research Institute for Aging, University of Waterloo, Waterloo, Ontario Canada; 6 Department of Health Research Methods, Evidence, and Impact, McMaster University, 1280 Main St West, Hamilton, ON Canada

**Keywords:** Nursing home, Long term care, Hip fracture, Mortality, InterRAI prediction

## Abstract

**Background:**

Frail older adults living in long term care (LTC) homes have a high fracture risk, which can result in reduced quality of life, pain and death. The Fracture Risk Scale (FRS) was designed for fracture risk assessment in LTC, to optimize targeting of services in those at highest risk. This study aims to examine the construct validity and discriminative properties of the FRS in three Canadian provinces at 1-year follow up.

**Methods:**

LTC residents were included if they were: 1) Adults admitted to LTC homes in Ontario (ON), British Columbia (BC) and Manitoba (MB) Canada; and 2) Received a Resident Assessment Instrument Minimum Data Set Version 2.0. After admission to LTC, one-year hip fracture risk was evaluated for all the included residents using the FRS (an eight-level risk scale, level 8 represents the highest fracture risk). Multiple logistic regressions were used to determine the differences in incident hip or all clinical fractures across the provinces and FRS risk levels. We examined the differences in incident hip or all clinical fracture for each FRS level across the three provinces (adjusted for age, BMI, gender, fallers and previous fractures). We used the C-statistic to assess the discriminative properties of the FRS for each province.

**Results:**

Descriptive statistics on the LTC populations in ON (*n* = 29,848), BC (*n* = 3129), and MB (*n* = 2293) are: mean (SD) age 82 (10), 83 (10), and 84 (9), gender (female %) 66, 64, and 70% respectively. The incident hip fractures and all clinical fractures for FRS risk level were similar among the three provinces and ranged from 0.5 to 19.2% and 1 to 19.2% respectively. The overall discriminative properties of the FRS were similar between ON (C-statistic = 0.673), BC (C-statistic = 0.644) and MB (C-statistic = 0.649) samples.

**Conclusion:**

FRS is a valid tool for identifying LTC residents at different risk levels for hip or all clinical fractures in three provinces. Having a fracture risk assessment tool that is tailored to the LTC context and embedded within the routine clinical assessment may have significant implications for policy, service delivery and care planning, and may improve care for LTC residents across Canada.

## Background

It has been estimated that 1 in 4 Canadians will be 65 years or older by 2036 [1]. As the population ages, a greater number of older adults will need residential support such as long-term care (LTC). LTC Residents are often frail, since their multiple physical and cognitive deficits place them at high risk of falls, disability, and death [2, 3]. Hip fractures are the most common type of fracture in LTC (49% of all fractures) [4]. They are more common in older adults living in LTC (49%) than in the community (29%) [4, 5], and lead to more hospitalizations [6] and worsening health-related quality of life [7]. In Canada, 45% of LTC residents with hip fracture die within 12 months [8] and of the survivors, 48% are no longer ambulatory [8].

Hip fracture prediction and prevention in LTC residents receive little attention due to the multiple comorbidities and medical complexity of LTC residents [9, 10] and the challenges of predicting fracture in this population. It is difficult to identify LTC residents with high fracture risk, as the commonly used fracture risk assessment tools in Canada, including the Canadian Fracture Risk Assessment Tool (FRAX) and the Canadian Association of Radiologists and Osteoporosis Canada tool (CAROC) [11–14], are not valid or generalizable for residents of LTC [15, 16]. FRAX and CAROC typically provide a 10-year fracture risk assessment timeframe, which is too long, given the mean 2.4-year life expectancy of LTC residents [17]. A recent study showed that FRAX (with bone mineral density) may predict incident hip fracture at one year [18]. Bone mineral density is heavily weighted in current fracture risk assessment protocols, but bone mineral density is not feasible to obtain in LTC. In addition, FRAX is not tailored to frail, institutionalized LTC residents. Thus, fracture prediction outputs of FRAX-Canada and CAROC may not be suitable for decision making and care planning among frail LTC residents [19, 20].

Recently, our team developed the Fracture Risk Scale (FRS) [21], a standardized outcome scale for identifying LTC residents at risk for fracture within one year. The FRS can be obtained from the Resident Assessment Instrument Minimum Data Set (RAI-MDS 2.0), which is a comprehensive, standardized assessment that is used upon admission and on a quarterly basis thereafter, to gather information on a wide range of socio-demographic and clinical characteristics [22–24]. The FRS was developed using Ontario residents’ data from the RAI-MDS 2.0, the Discharge Abstract Database (DAD) and the National Ambulatory Care Reporting System (NACRS). However, the FRS was not externally validated (in a population other than the tool development sample), nor was its validity tested in other Canadian provinces. As a predictive model, the FRS’ reproducibility (performing sufficiently accurate across new samples from the same target population) and transportability (performing well across samples from different but related source populations) [25] need to be tested prior widespread adoption. Therefore, we conducted this validity study to examine the FRS performance across a new sample and in different but related population of LTC residents in other provinces [21].

This study aims to: 1) assess the discriminative properties of the FRS using C-statistics and examine the construct validity of the FRS by comparing incident hip fractures and all clinical fractures (includes hip, spine, humerus, forearm, pelvis fractures) for each fracture risk levels in LTC residents across three Canadian provinces at 1-year follow up; 2) compare incident hip and all clinical fractures in LTC residents across three Canadian provinces; and 3) compare incident hip and all clinical fractures between the FRS risk levels. To examine the construct validity study of the FRS [26], we hypothesize that incident hip fractures for each fracture risk levels in LTC residents in the three Canadian provinces are not statistically different when type 1 error is ≤0.05.

## Methods

### Study design and population

This retrospective cohort study uses data from the RAI-MDS 2.0. In Canada, LTC facilities provide living accommodation for people who need 24-h professional health services, personal care and services, including meals, laundry, housekeeping, frequent assistance with activities of daily living, on-site supervision or monitoring to ensure safety or well-being [27, 28]. LTC residents were included if they: 1) were adults admitted to LTC homes in Canada from April 1st, 2006 to March 31st, 2010; and 2) were assessed with the RAI-MDS 2.0. LTC residents were excluded if they: 1) had multiple admissions; 2) reported on the RAI-MDS 2.0 to have end stage disease, were comatose, received hospice, or respite care; 3) were expected to have a short stay; 4) had the admission assessment completed more than 14 days after the date of admission (to exclude any assessment for existing residents); or 5) had no reassessments during the one-year follow-up. The project received ethics approval from the University of Waterloo Office of Research Ethics (ORE no 17045).

### Fracture rating scale

The FRS is different from existing fracture risk assessment tools in that it does not use bone mineral density and includes fracture risk factors that are relevant to the long-term care (LTC) population [21]. Moreover, to ensure that it was valid for LTC residents and easily scalable, the FRS was designed and validated using large population-based datasets that include routinely collected data from LTC residents. The FRS was developed using decision tree analysis and the included items are walking in corridor (independent, supervision to extensive assistance or total assistance/walking did not occur), wandering (no wandering to infrequent wandering, less than daily wandering, or daily wandering), falling status within the past 30 days (no/yes), cognitive performance scale (intact cognition, borderline intact or mild impairment, or moderate to very severe impairment), transfer status (how resident moves between surfaces to and from; bed, chair, wheelchair, standing position), (independent to extensive assistance, or total assistance/transfer did not occur), age greater than 85 years (no/yes), body mass index (BMI) (< 18, 18–30, or > 30 kg/m^2^), and previous fractures in past 180 days (no/yes) [21]. The FRS includes eight fracture risk level categories (level 1 represents the lowest and level 8 represents the highest fracture risk). Within the FRS, assessment of risk continues through the decision tree until a terminal risk level is identified. For example, individuals who can walk in corridor independently with BMI > 30 are defined as fracture risk level 3. However, if BMI is between 18 and 30, risk level also depends on history of falls, fracture and cognitive performance.

### Incident fractures

Over the course of one-year follow-up period, residents were classified as to the presence or absence of an incident fracture. To capture incident fractures, we accessed in-patient hospital records and emergency department records [23, 24]. DAD is received directly from acute care facilities or their respective health/regional authority or ministry/department of health and it contains demographic, administrative and clinical data of all Canadian provinces except Quebec [29]. NACRS contains data for all hospital-based and community-based ambulatory care including day surgery, outpatient and community-based clinics and emergency departments [30]. NACRS data is received directly from participating facilities or from regional health authorities or ministries of health. Data collection methods may vary by facility. We were able to link DAD and NACRS data to RAI-MDS data for this analysis.

Incident fractures were defined using International Statistical Classification of Diseases and Related Health Problems, 10th Revision, Canada (ICD-10-CA) codes, captured in DAD and NACRS. The codes were selected using the Revised Framework for National Surveillance on Osteoporosis and Osteoporosis-related Fractures of the Public Health Agency of Canada [31]. A resident with one of the hip fracture codes (hip (S72.0, S72.1, S72.2) present on either a hospitalization or emergency department visit within one year after the admission assessment was code as having hip fracture. A resident with at least one of these codes within one year after the admission assessment was coded as having a fracture (hip (S72.0, S72.1, S72.2), spine (S22.0, S22.1, S32.0, S32.7, S32.8), humerus (S42.2), forearm (S52.x, S62.x) and pelvis (S32.1, S32.3, S32.4, S32.5, S32.7, S32.8)).

### Statistical analysis

The study population demographics, prior falls and fractures, and fracture incidence estimates are expressed as mean (SD) for continuous data and counts and percentages for categorical data. Percent of incident hip fracture and all clinical fractures (hip, spine, humerus, forearm, pelvis fractures) for each FRS risk level in the three provinces were calculated. Multivariable logistic regression models were used to determine if incident hip fractures are different across the provinces and to calculate the odds ratio of incident hip fractures in each FRS risk level. In this logistic regression, the incident hip fractures were the dependent variable (DV) and provinces (Ontario was used as the reference group) and FRS risk levels (FRS risk level 1 was used as the reference level) were the independent variables (IV). We tested the significance of the interaction term of FRS and provinces in the logistic regression model to determine if the incident hip fracture in each FRS risk level is different across the provinces. The logistic regression analyses were adjusted for age, BMI, gender, fallers in the last 180 days, previous fracture, and the size of the residential home (small, medium or large). All the analyses were repeated using all incident fractures as DV. To assess the discriminative properties of the FRS for each province, we used the C-statistic. All the statistical analyses were conducted using SAS V.9.4 (SAS Institute).

## Results

**Fig. 1:**
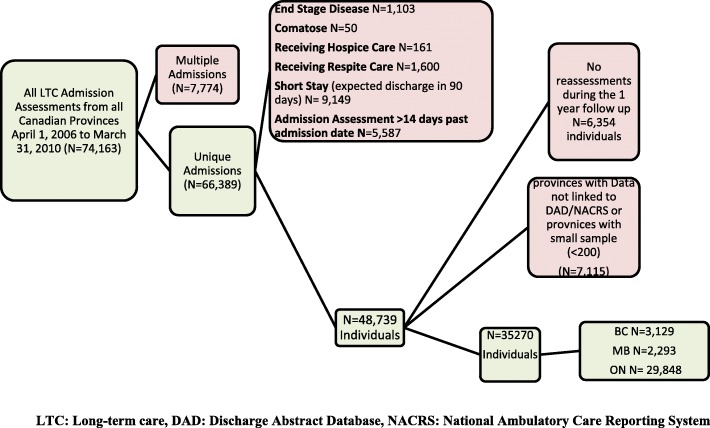
Study sample flow diagram

The final study sample includes 35,270 participants (ON = 29,848, BC = 3129 and MB = 2293) and is displayed in Fig. 1. Of the eligible residents, 703 (ON = 447, BC = 169 and MB = 87) were not included in the analysis due to data entry errors. Table 1 demonstrates the characteristics of LTC residents in the three provinces. Table 2 describes FRS items in LTC residents of the three provinces. Of the included residents, 3561 (10.1%) residents died before the end of the one-year follow-up period. The characteristics of the excluded and included resident are comparable. Of the excluded residents 48.8% aged 85 years or older, and 67.9% are female. The age, gender distribution and comorbidities are similar across the provinces; with less than 1% of LTC residents having had a hospital admission within 3 months from LTC admission. Falls, incident fractures, and BMI were similar across the provinces as well. All of the Manitoba homes in the study were from one urban centre, as the remainder of the province does not use RAI-MDS.

**Table 1:** Baseline characteristics of the study participants

Variables	ON (*N* = 29,848)	BC (*N* = 3129)	MB (*N* = 2293)
Age, yrs. Mean (SD)	82.1 (9.6)	83.0 (9.6)	83.5 (9.3)
Gender, Female, *n* (%)	19,706 (66.1)	2023 (64.7)	2293 (69.8)
Married, *n* (%)	8978 (30.1)	818 (26.2)	608 (26.5)
Chronic Disease Number, *n* (%)			
Osteoporosis	7247 (24.3)	663 (21.2)	353 (15.4)
Diabetes Mellitus	7239 (24.3)	610 (19.5)	472 (20.6)
Arthritis	10,486 (35.1)	1058 (33.8)	811 (35.4)
Alzheimer’s Disease	5513 (18.5)	523 (16.7)	289 (12.6)
Cancer	2935 (9.8)	280 (9.0)	279 (12.2)
Neuromuscular diseases	47 (0.2)	11 (0.4)	1 (0.04)
Parkinson’s Diseases	81 (0.3)	2 (0.1)	6 (0.3)
Liver Disease	69 (0.2)	7 (0.2)	7 (0.3)
Arteriosclerotic Diseases	2004 (6.7)	199 (6.4)	127 (5.5)
Hospital admissions in past 90 days, *n* (%)			
No Visit	29,603 (99.2)	3106 (99.3)	2281 (99.5)
≥ 1 Visit	245 (0.8)	23 (0.7)	12 (0.5)
Emergency room visits in past 90 day,s *n* (%)			
No Visit	26,590 (89.1)	2927 (93.5)	2232 (97.3)
≥ 1 Visit	3258 (10.9)	202 (6.5)	61 (2.7)
Number of prescribed medications, Mean (SD)	9.7 (4.63)	8.45 (4.04)	8.02 (4.66)
Changes in Health, End-Stage Disease, Signs, and Symptoms (CHESS Score), *n* (%)			
0	110,18 (58.69)	1309 (68.64)	664 (72.49)
1	4665 (24.85)	362 (18.98)	188 (20.52)
2	2179 (11.61)	176 (9.23)	51 (5.57)
3	692 (3.69)	49 (2.57)	11 (1.20)
4	219 (1.17)	11 (0.58)	2 (0.22)
5	0	0	0
Home size, *n* (%)			
Small (≤50 beds)	583 (1.95)	276 (8.82)	0
Med (51–99 beds)	511,9 (17.15)	103,7 (33.14)	278 (12.12)
Large (≥ 100 beds	24,146 (80.90)	181,6 (58.04)	2015 (87.88)
Overall case mix index of the residents at Baseline, Mean (SD)	0.64 (0.18)	0.57 (0.16)	0.58 (0.14)
Ownership, *n* (%)			
Public/religious/not for profit	127,41 (42.69)	157,3 (50.27)	122,3 (53.34)
Private	171,07 (57.31)	155,6 (49.73)	107,0 (46.66)
Rurality, *n* (%)			
Urban	255,96 (85.75)	272,3 (87.02)	2293 (100)
Rural	410,5 (13.75)	401 (12.82)	0

**Table 2:** Fracture rating scale items in the three provinces

Fracture rating scale items	ON (*N* = 29,848)	BC (*N* = 3129)	MB (*N* = 2293)
Age group			
18 to 64	1700 (5.7)	162 (5.2)	92 (4.0)
65 to 74	3128 (10.5)	325 (10.4)	198 (8.6)
75 to 84	11,300 (37.9)	1028 (32.9)	799 (34.9)
85+	13,708 (45.9)	1613 (51.6)	1202 (52.5)
Body Mass Index			
< 18	2396 (8.0)	423 (13.5)	254 (11.1)
18–29	22,252 (74.6)	2310 (73.8)	1677 (73.1)
30+	4753 (15.9)	396 (12.7)	362 (15.8)
Wandering Frequency			
Not in last 7 days	22,825 (76.5)	2340 (74.8)	1856 (80.9)
1 to 3 days (in past 7 days)	1925 (6.5)	275 (8.8)	141 (6.2)
4 to 6 days (in past 7 days)	1614 (5.4)	197 (6.3)	83 (3.6)
Daily (in past 7 days)	3484 (11.7)	317 (10.1)	213 (9.3)
Walking in corridor			
Independent	10,530 (35.3)	1500 (47.9)	1016 (44.3)
Supervision	4477 (15.0)	294 (9.4)	223 (9.7)
Limited assistance	2789 (9.3)	223 (7.1)	197 (8.59)
Extensive assistance	2086 (7.0)	107 (3.4)	74 (3.2)
Total dependence	381 (1.3)	27 (0.9)	35 (1.5)
Activity did not occur	9585 (32.1)	978 (31.3)	748 (32.6)
Transfer status			
Independent	9569 (32.1)	1510 (48.3)	1054 (46.0)
Supervision	3576 (12.0)	254 (8.1)	190 (8.29)
Limited assistance	4662 (15.6)	468 (15.0)	411 (17.9)
Extensive assistance	7140 (23.9)	481 (15.4)	255 (11.1)
Total dependence	4806 (16.1)	396 (12.7)	382 (16.7)
Activity did not occur	95 (0.3)	20 (0.6)	1 (0.04)
Cognitive performance scale			
Intact	5159 (17.3)	400 (12.8)	258 (11.3)
Borderline Intact	4517 (15.1)	478 (15.3)	370 (16.1)
Mild	6270 (21.0)	657 (21.0)	440 (19.2)
Moderate to very severe	8697 (29.1)	1037 (33.1)	802 (35.0)
Moderate Severe	1650 (5.5)	143 (4.6)	104 (4.5)
Severe	2538 (8.5)	354 (11.3)	256 (11.2)
Very Severe	1017 (3.4)	60 (1.9)	63 (2.8)
Falls within the last 30 days, *n* (%)			
No Falls	24,620 (82.5)	2569 (82.1)	1923 (83.9)
≥ 1 Fall	5228 (17.5)	560 (17.9)	370 (16.1)
Prior hip fracture in last 180 days, *n* (%)	179,088 (6)	18,774 (6)	6879 (3)

**Fig. 2:**
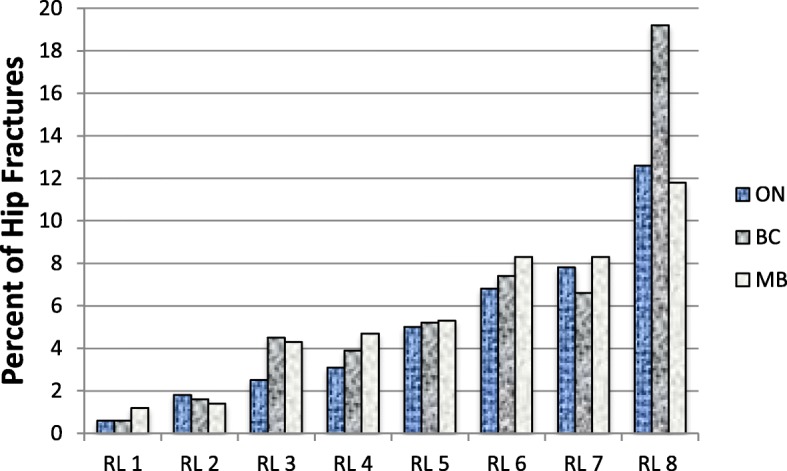
Incident Hip fracture for Fracture Rating Scale risk levels

**Fig. 3:**
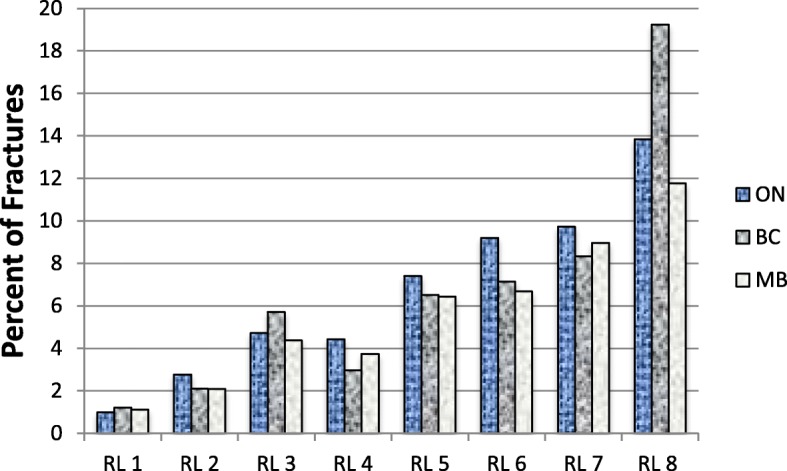
All Incident fracture for Fracture Rating Scale risk levels

Incident hip fractures and all clinical fractures in all risk levels ranged from 0.5 to 19.2% and 1 to 19.2% respectively (Figs. 2 and 3, respectively). Table 3 demonstrates the provincial fracture counts and percent by FRS levels. The incident hip fractures were comparable across the three provinces (BC = 1.27 (1.04, 1.55), MB = 1.30 (1.04, 1.62), when ON is the reference province). Also, there was no statistically significant difference in all clinical fractures (BC = 0.97 (0.81, 1.16), MB = 0.84 (0.68, 1.04), when ON is the reference province) across the three provinces (Table 4). When adjusting for the provinces, the odds of incident hip fractures and all clinical fractures in all the FRS risk levels were significantly different compared to level 1 with consistently increasing odds of fractures with higher FRS risk levels, as shown in Table 4.

**Table 3:** Provincial fracture counts and percent by FRS levels

	Hip Fracture *n* (%)			All Fractures *n* (%)		
FRS risk levels	ON	BC	MB	ON	BC	MB
1	15 (0.5)	2 (0.8)	3 (1.1)	30 (1.0)	3 (1.2)	3 (1.1)
2	53 (1.8)	5 (1.5)	4 (1.7)	82 (2.8)	7 (2.1)	5 (2.1)
3	194 (2.6)	39 (4.4)	29 (4.2)	353 (4.7)	51 (5.7)	30 (4.4)
4	174 (2.5)	13 (2.4)	14 (3.7)	305 (4.4)	16 (3.0)	14 (3.7)
5	238 (4.6)	31 (5.2)	21 (5.4)	380 (7.4)	39 (6.5)	25 (6.4)
6	47 (6.8)	2 (7.1)	1 (6.7)	64 (9.2)	2 (7.1)	1 (6.7)
7	205 (6.7)	20 (6.9)	17 (8.0)	296 (9.7)	24 (8.3)	19 (9.0)
8	20 (12.6)	5 (19.2)	2 (11.8)	22 (13.8)	5 (19.2)	2 (11.8)
Total^a^	959 (3.2)	124 (4.0)	92 (4.0)	1553 (5.2)	154 (4.9)	100 (4.4)

^a^= Total fractures do not add up due to excluding participants with data entry error

**Table 4:** Differences in Hip Fracture across provinces and Fracture risk scale risk levels

Variables	Hip Fractures	All Fractures
	OR (CI)	OR (CI)
Provinces		
ON	1	1
BC	1.27 (1.04, 1.55)	0.97 (0.81, 1.16)
MB	1.30 (1.04, 1.62)	0.84 (0.68, 1.04)
FRS risk levels		
1	1	1
2	3.08 (1.86, 5.11)	2.64 (1.79, 3.89)
3	5.56 (3.52, 8.80)	5.28 (3.74, 7.45)
4	4.29 (2.68, 6.92)	3.84 (2.68, 5.50)
5	8.02 (5.06, 12.72)	6.93 (4.90, 9.81)
6	12.17 (7.15, 20.73)	8.97 (5.90, 13.65)
7	10.52 (6.60, 16.79)	8.36 (5.86, 11.91)
8	17.00 (10.15, 35.55)	10.90 (6.41, 18.59)

*OR* Odds Ratio, *CI* 95% Confidence Interval, *ON* Ontario, *BC* British Colombia, *MB* Manitoba, *FRS* Fracture Rating Scale. The analyses were adjusted for age, BMI, gender, fallers in the last 180 days, previous fracture, and Home size (small, medium or large)

All the interaction terms associated with FRS risk levels and provinces were not statistically significant. This indicates the similarity of incident hip fractures and all clinical fractures for each FRS risk levels in the three provinces. The overall discriminative properties of the FRS were similar between ON (c-statistic = 0.67), BC (c-statistic = 0.64) and MB (c-statistic = 0.65) samples. The proportion of hip fractures that occurred in residents with a FRS level of 1–3 ranged from 4.9–7% across provinces, whereas the proportion of hip fractures that occurred in residents with a FRS of 6–8 was higher (> 26%).

## Discussion

Our study demonstrates the validity of the FRS for predicting hip and all clinical fractures in LTC residents living in several Canadian provinces. As recommended by the Cosmin initiative [26], an international initiative that aims to improve the selection of health measurement instruments; hypothesis-testing construct validity is a critical component of evaluating outcome measures’ psychometric properties. This study confirmed our hypothesis and showed that incident hip and all clinical fractures for each FRS risk levels in LTC residents in the three Canadian provinces were similar, confirming that the FRS is reproducible and transportable for LTC residents living in different geographical areas [25]. Therefore, our results confirmed that FRS can be used to identify LTC residents with high risk of hip or all fractures across different Canadian provinces.

We have previously shown that the FRS is able to identify LTC residents at highest risk of fracture in the initial FRS development study [21]. The FRS is an adequate reflection of the outcome of interest (one-year incident fracture). Our study builds on this work by examining FRS performance in LTC residents in three geographical areas. A clinical prediction tool performance is determined by assessing a model’s calibration and discrimination [32]. Calibration is the agreement between prediction from the model (FRS model) and observed outcomes (incident hip and all clinical fractures) and indicates a model’s predictive accuracy [33], which was shown in Table 4. Discrimination is the ability of the prediction model (FRS model) to set apart participants with and without the outcome of interest (incident hip and all clinical fractures); those with the outcome of interest should have a higher predicted risk compared to those who do not have it [33] which was also shown in Table 4.

Unlike commonly used fracture risk assessment tools (FRAX and CAROC), FRS is a unique one-year fracture prediction tool that is composed of risk factors specific to LTC residents. FRS is embedded in the RAI-MDS 2.0, which is completed within 14 days of a resident entering a home and quarterly thereafter. In addition, the FRS can be obtained from the interRAI Long Term Care Facility assessment, which is the successor to the RAI-MDS 2.0 [24, 34]. Thus, FRS can be easily and regularly implemented in LTC without burden on the LTC staff [9, 10, 35, 36]. As we demonstrated the FRS’ validity, we suggest it can be used for LTC resident care planning across Canada and possibly internationally. Recently, Fracture Risk Assessment in Long-term Care (FRAiL) tool has been developed, which aims to predict two-year hip fracture risk in nursing home residents [16]. The FRAiL tool developed using RAI-MDS 2.0 assessment of nursing home residents in the United States. FRAiL tools included Fifteen items to predict hip fracture: older age, white race, female, impaired cognition, activities of daily living independence, locomotion independence, urinary continence, previous falls, transfer independence, easily distracted, wandering, absence of osteoarthritis, absence of pressure ulcer, low BMI, and diabetes. There are some common items in FRS and FRAiL tools such as wandering, falling, cognitive performance scale, transfer status, age, BMI, and previous fractures.

Recommendations for preventing fracture in LTC have been developed to provide non-pharmacologic and pharmacologic strategies for fracture prevention in frail older adults living in LTC [37]. However, there is a current gap in osteoporosis treatment and fracture prevention in LTC residents [38–43]. One of the barriers to implement fracture prevention guideline is the lack of information about fracture risk assessment [44]. The easily implemented and validated FRS tool may overcome this barrier. To help LTC clinicians prescribing the appropriate intervention to residents who are identified as “at risk”, our team will develop and implement an electronic Clinical Assessment Protocol (CAP) in LTCs. The Fracture Risk CAP will automatically produce recommendations for residents based on their FRS fracture risk level and will inform clinical decision making as part of the person-centered care planning process to fill the gap of fracture prevention in LTC. Other CAPs have been developed to draw the attention of the healthcare provider to a matter (such as Activities of daily living, delirium and Cardiorespiratory) that can be improved and should be considered in LTC residents’ care plan [45].

Strengths of this study include the use of a large, representative sample of LTC residents with RAI-MDS 2.0 data linked with DAD and NACRS. As it is recommended to externally validate the model in samples from related source populations (LTC residents in other provinces) [25], we examined the performance of the FRS outside of the development sample. Assessing the relation between the development and validation samples allowed us to explain the FRS (clinical) transportability and (statistical) reproducibility [25].

We acknowledge that our study has limitations. We excluded LTC residents with short life expectancy based on the data from the RAI-MDS 2.0 (participants who have end stage disease, were comatose, received hospice, or respite care, or expected a short stay), as we aim to inform decisions related to fracture prediction and prevention for those whom life expectancy is 1 year or greater. Therefore, our findings may not be generalizable to some LTC residents. We included participants who died before the end of the one-year follow up in the analysis, which may underestimate the incident fracture, as these people may have a higher likelihood of fracture. Competing risk framework was not employed in the analyses, as we excluded individuals who were likely to die within a year apriori. Despite not employing competing risk framework, our result shows that FRS performs reasonably well in identifying at risk people. We used a validated framework to capture incident fractures (Revised Framework for National Surveillance on Osteoporosis and Osteoporosis-related Fractures of the Public Health Agency of Canada [31]), however, vertebral and other fractures are likely not fully captured. Our study was limited to the variables collected in the RAI-MDS 2.0 and may not have captured all relevant risk factors for hip or all clinical fractures in LTC residents.

## Conclusion

The FRS is a valid tool for identifying LTC residents at different risk levels for hip or all clinical fractures in three provinces. Having a fracture risk assessment tool that is tailored to the LTC context and embedded within the routine clinical assessment may have significant implications for policy, service delivery and care planning, and may improve care for LTC residents across Canada.

## Abbreviations

BC: British Columbia
CAP: Clinical Assessment Protocol
CAROC: Canadian Association of Radiologists and Osteoporosis Canada tool
CI: Confidence Interval
DAD: Canadian Discharge Abstract Database
DV: Dependent Variable
FRAX: Fracture Risk Assessment Tool
FRS: Fracture Rating Scale
ICD-10 code: International Classification of Disease-10 code
IV: Independent Variable
LTC: Long Term Care
MB: Manitoba
NACRS: National Ambulatory Care Reporting System
ON: Ontario
RAI-MDS 2.0: Resident Assessment Instrument Minimum Data Set
SD: Standard Deviation
